# Prescribing Z-drugs in Greece: an analysis of the national prescription database from 2018 to 2021

**DOI:** 10.1186/s12888-023-04793-x

**Published:** 2023-05-26

**Authors:** Spyridon Siafis, Konstantinos N. Fountoulakis, Vasileios Fragkidis, Georgios Papazisis

**Affiliations:** 1 Department of Psychiatry and Psychotherapy, School of Medicine, Technical University of Munich, Munich, Germany; 2 Department of Clinical Pharmacology, School of Medicine, Faculty of Health Sciences, Aristotle University of Thessaloniki, Thessaloniki, Greece; 3 3rd Department of Psychiatry, School of Medicine, Faculty of Health Sciences, Aristotle University of Thessaloniki, Thessaloniki, Greece; 4 Clinical Research Unit, Special Unit for Biomedical Research and Education, School of Medicine, Faculty of Health Sciences, Aristotle University of Thessaloniki, Thessaloniki, Greece

**Keywords:** Sleep disorders, Pharmacoepidemiology, Z-drugs, Zolpidem, Zopiclone, Greece

## Abstract

**Background:**

The Z-drugs are indicated for the short-treatment of insomnia, but they are associated with abuse, dependence and side-effects. There are only sparse data about Z-drug prescribing in Greece.

**Methods:**

We analyzed data from the Greek prescription database, considering prescriptions for the available Z-drugs in Greece, i.e., zolpidem and zopiclone, during the period from 01.10.2018 to 01.10.2021 in order to examine the prevalence, monthly number and characteristics of Z-drug prescriptions in Greece.

**Results:**

There were 1,229,842 prescriptions for Z-drugs (zolpidem: 89.7%) during the investigated period from 2018 to 2021, which corresponded to 156,554 patients (73.1% ≥ 65 years, 64.5% female). More than half of the patients (65.8%) had more than one prescription with a median number of 8, interquartile range IQR [3, 17], prescriptions during the three-year study period. Most patients (76.1%) were prescribed by medical specialties other than psychiatrists and neurologists, despite a considerable frequency of psychiatric comorbidities (53.7%). About half of patients with anxiety/depression were not prescribed anxiolytics or antidepressants, a practice more frequently observed among medical specialties other than psychiatrists and neurologists. The average annual prevalence of at least one prescription for Z-drugs in the Greek population during 2019–2020 was approximately 0.9% (higher in females and older adults). The monthly number of prescriptions was relatively stable with a median number of 334.2 IQR [310.4; 351.6] prescriptions per 100,000 persons.

**Conclusions:**

A considerable number of patients are prescribed Z-drugs in Greece, more often older adults, females and patients with psychiatric comorbidities. The prescribing physicians were in the majority (70%) internists and general practitioners, while psychiatrists (10.9%) and neurologists (6.1%) accounted for a smaller proportion. Due to the limitations inherent to medical claims databases, further research is warranted in order to elucidate the potential abuse and misuse of Z-drugs.

**Supplementary Information:**

The online version contains supplementary material available at 10.1186/s12888-023-04793-x.

## Background

Insomnia is a common mental disorder, affecting approximately 7% of the population in Europe and resulting in a significant burden of 94 disability-adjusted life years (DALYs) per 100,000 persons [1]. In Greece, the prevalence is unclear, and estimates of self-reported insomnia have ranged from approximately 25% in 2010 [2] to 40% during the first wave of COVID-19 pandemic in 2020 [3].

Although cognitive-behavioral therapy for insomnia (CBT-I) is the recommended first-line treatment, it is not widely available and may not be effective for all individuals [4]. Thus, pharmacological interventions are frequently used, with a wide range of effective drugs available that are generally divided into two main categories: the benzodiazepine receptor agonists, i.e., benzodiazepines and non-benzodiazepines (Z-drugs), and other drugs, e.g., melatonin, and orexin antagonists [4, 5]. The prescription patterns of Z-drugs are particularly of great importance, as these medications were initially marketed and perceived as safe alternatives to benzodiazepines, yet they can also result in abuse and dependence, among other side-effects [4, 6].

The prevalence of Z-drug prescriptions can vary significantly across different countries and regions [7–11], which makes it challenging to make generalizations. There are generally only sparse data about the situation in Greece. The implementation of a nationwide electronic prescription system during the 2010s represented a significant shift from paper-based to digital prescriptions for medications. However, it was not mandatory to issue electronic prescriptions for medications with abuse potential, including Z-drugs, until 17.07.2019. As a result, the current study aims to analyze prescriptions for Z-drugs from the Greek nationwide prescription database, with the aim to investigate the prevalence of Z-drug prescriptions, their characteristics and trends.

## Material and methods

### Study design

This was a retrospective pharmaco-epidemiological study using the Greek nationwide prescription database.

### Dataset

Anonymized data from the Greek nationwide prescription database were used. This database is managed by the Greek e-Government Center for Social Security Services (IDIKA S.A.) and covers almost the entire Greek population (97–98%, except people without a social security number) [12].

The dataset contained information about the age, sex, prescribed medications (classified using the Anatomical Therapeutic Chemical Classification, ATC), diagnosis (classified with International Classification of Diseases Tenth Revision, ICD-10), the month dispensed by the pharmacies, the geographical region, and the specialty of the prescribing physician. No information was obtained about the prescribed dose and dosing schedule, except for the number of prescribed boxes. We obtained data for prescriptions of zolpidem (ATC: N05CF02) and zopiclone (ATC:N05CF01). Zaleplon and eszopiclone were not considered, since they were withdrawn by the European Medicines Agency and subsequently from the Greek market. Although zolpidem and zopiclone have an official indication of insomnia, the electronic prescription system permits the prescription for Z-drugs through the use of ICD-10 codes not only for sleep disorders (ICD: G47 and F51), but also for other psychiatric (ICD-10: F00-F99) and neurological disorders (ICD-10: G00-G99).

In Greece, prescriptions are issued by the physicians and subsequently dispensed by the pharmacies. Therefore, we considered only dispensed prescriptions. We were unable to obtain any data for prescriptions other than those containing Z-drugs among patients who were prescribed them. This may have resulted in gaps in our dataset concerning co-morbidities and co-prescribed medications. The investigated timeframe was between 01.10.2018 and 01.10.2021.

### Data analysis

#### Differences between prescriptions by psychiatrists or neurologists in comparison with other specialists

We examined differences between prescriptions by psychiatrists or neurologists and by other medical specialties using a t-test or Mann–Whitney U test for continuous variables and a chi-squared test and odds ratios (OR) for dichotomous variables.

#### Prevalence estimations of Z-drug prescription

The prevalence of patients with a least one Z-drug prescription was estimated using the population data from the Hellenic Statistical Authority as the denominator [13]. In particular, we calculated the overall age- and sex-standardized annual prevalence for each year (2019, 2020) separately, and also determined the weighted average annual prevalence over the period of 2019–2020. We also calculated the annual prevalence stratified by age group and sex. Differences in the prevalence between the two years were quantified with ORs. It is worth noting that the prevalence was not estimated for 2018 and 2021, as data for these years were only available for certain periods.

#### Prescription trends and the effect of the electronic prescription mandate

We examined the monthly trends of prescriptions within the three-year investigated timeframe (01.10.2018–01.10.2021). We standardized the monthly number of prescriptions per 100,000 persons by dividing them with the estimated average total population of Greece between 2018 and 2021, which was 10,715,740.

There were two important dates within this timeframe: i) the date of the electronic prescription mandate on 17.07.2019, after which the prescriptions for zolpidem and zaleplon should be electronically registered to the database, ii) the date of the first COVID-19 case in Greece on 26.02.2020. In addition, there was an unexpected shortage of Z-drugs in Greece during the period roughly from April to August 2021. The monthly number of prescriptions before and after the electronic mandate was compared with a Mann–Whitney U test, but excluding the time period after the shortage of Z-drugs (i.e., 10.2018–07.2019 vs. 08.2019–03.2021). Data analysis was conducted in R statistical software v.4.0.3 [14].

## Results

### Prescription characteristics

During the investigated period of three years, 1,229,842 prescriptions for Z-drugs (zolpidem:89.4%, zopiclone: 10.4%, both: 0.3%) were dispensed by pharmacies in Greece, which corresponded  to 156,554 unique patients.

Among the total prescriptions, 69.7% were in female patients, and the median age of the patients was 77 years. The most frequently diagnoses are presented in the eAppendix-1.1. The most prevalent diagnoses were sleep disorders (ICD-10: G47) in 30.5% and non-organic sleep disorders (ICD-10: F51) in 21.8% of the prescriptions eAppendix-1.2.

In addition, 51.8% of the prescriptions had at least one co-prescribed medication, such as bromazepam (12.8%), alprazolam (11.6%) and paracetamol (7.5%) (eAppendix-1.3). In the prescriptions that had a diagnosis of anxiety or depressive disorder, about half of them had not a co-prescribed anxiolytic or antidepressant.

About half of the prescriptions concerned patients in Attika (33.5%) or Thessaloniki (9.1%) (these two regions account for about half of the population in Greece) (eAppendix-1.4). More than half of the patients (65.8%) had more than one prescription for Z-drugs, with a median number of 8, IQR [3,17] prescriptions, during the three-year study period. The characteristics of patients are presented in Table 1.

**Table 1:** Characteristics of the 156,554 individuals who were prescribed at least once zolpidem and/or zopiclone from 01.10.2018 to 01.10.2021 in Greece

Variable		N of patients	Frequency (%) (out of 156,554 individuals unless otherwise reported) / median, interquartile range [IQR]
**Z-drug**	**Zolpidem (ATC: N05CF02)**	130,815	83.6%
	**Zopiclone (ATC: N05CF01)**	13,846	8.8%
	**Both**	11,893	7.6%
**Age**	**Age in years**		75 years, IQR [63; 84]
	**≥65 years old**	114,472	73.1%
**Sex**	**Female**	101,045	64.5%
	**Male**	55,509	35.5%
**Region**	**Attika**	54,297	34.7%
	**Thessaloniki**	14,825	9.5%
	**Other**	87,432	55.8%
**Sleep disorders**	**Sleep disorders (ICD-10: G47)**	63,861	40.8%
	**Nonorganic sleep disorders (ICD-10: F51)**	49,257	31.5%
	**Any sleep disorder (ICD-10: G47 and/or F51)**	100,406	64.1%
**Psychiatric disorder (other than sleep disorder)**	**Depression (ICD-10: F32, F33)**	19,795	12.6%
	**Anxiety (ICD-10: F40, F41)**	61,112	39.0%
	**Anxiety and/or depression (ICD-10: F32, F33, F40, F41)**	72,894	46.6%
	**Any psychiatric disorder (ICD-10: F1-F9; except dementia and sleep disorder)**	84,034	53.7%
**Co-prescribed medications (ATC)** ^**a**^	**Opioid analgesics (ATC: N02A)**	4959	3.2%
	**Nonopioid analgesics (ATC: N02B)**	17,491	11.2%
	**Antiepileptics (ATC: N03A)**	5161	3.3%
	**Antipsychotics (ATC: N05A)**	8419	5.4%
	**Anxiolytics (ATC: N05B)**	52,992	33.8%
	**Antidepressants (ATC: N06A)**	21,025	13.4%
	**Any co-prescribed medication (any ATC)**	120,589	77.0%
**No co-prescribed anxiolytics and/or antidepressants in individuals with anxiety and/or depression (i.e., 72,894)** ^**a**^		33,094	45.4%
**Prescribed physician**	**Prescriptions only by psychiatrists or neurologists**	23,598	15.1%
	**At least one prescription by psychiatrist or neurologists**	13,863	8.9%
	**Prescriptions only be medical specialties other than psychiatrists and neurologists**	119,093	76.1%
**Frequency of prescriptions for Z-drugs within the three-year period**	**Number of patients with more than one prescription**	103,082	65.8%
	**Number of prescriptions in patients with more than one prescriptions (in 53,472 individuals)**		8 prescriptions, IQR [3,17]

Characteristics of the 156,554 individuals who were prescribed at least once zolpidem and/or zopiclone from 01.10.2018 to 01.10.2021 in Greece. ICD-10 and co-prescribed medications (ATC codes) that were reported in at least one of the prescriptions of the patients. The denominator in the frequencies was 156,554, unless otherwise reported

^a^We did not have access to data for all prescriptions, i.e., prescriptions that did not contain Z-drugs, thus, the frequency of co-prescription with other medications may be underestimated

### Prescribing physicians

The prescribing physicians were in the following order: internists (36.6% of the prescriptions), general practitioners (33.4%), psychiatrists (10.9%), neurologists (6.1%), cardiologists (5.3%), resident physicians in training (4.5%), nephrologists (0.9%), and other medical specialists in the rest 2.4% of the prescriptions.

There were differences between prescriptions by psychiatrists or neurologists and by other medical specialists (eAppendix-2). Prescriptions by psychiatrists or neurologists were on average in younger patients (median 69 years vs. 78 years), less frequently in females (65.9% vs. 70.4%) and less frequently having zolpidem as the Z-drug (85.4% vs. 90.5%). They had also less frequently an indication of sleep disorder (27.9% vs. 57.2%). In addition, they had more frequently a co-prescription for anxiolytics (45.7% vs. 27.9%), antidepressants (14.2% vs. 6.3%), antipsychotics (8.8% vs. 1.9%) and antiepileptics (3.3% vs. 1.5%), but less frequently for non-opioid (2.8% vs. 8.4%) and opioid analgesics (0.6% vs. 1.9%). In the prescriptions that had a diagnosis of anxiety or depressive disorder, co-prescription with anxiolytic or antidepressant was more frequent by psychiatrists or neurologists compared with other medical specialists (63.1% vs. 44.6%).

### Prevalence of Z-drug prescription in Greece

**Fig. 1:**
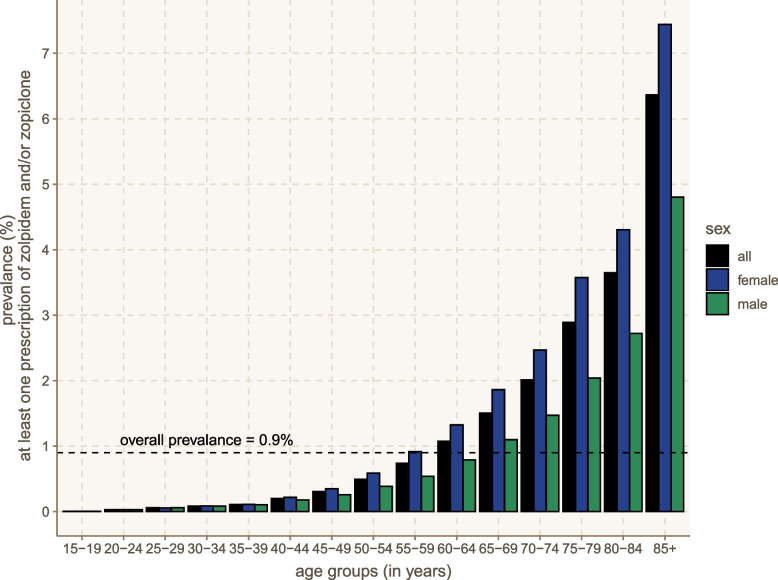
Prevalence of Z-drug prescription in Greece. The figure presents the weighted average annual prevalence over 2019–2020 stratified by age group and sex

The estimated annual prevalence of patients with at least one Z-drug prescription in the Greek population was 0.85% in 2019 and 0.89% in 2020 (standardized by age group and sex). The prevalence was higher in female patients and exponentially increased with the age reaching up to about 7% in > 85 years old. In addition, the prevalence increased in 2020 compared to 2019, particularly among younger age groups, yet a decrease was noted among individuals over the age of 85 years (eAppendix-3). The weighted average of the annual prevalence over 2019–2020, stratified by age group and sex, is presented in Fig. 1.

### Prescription trends and the electronic prescription mandate

**Fig. 2:**
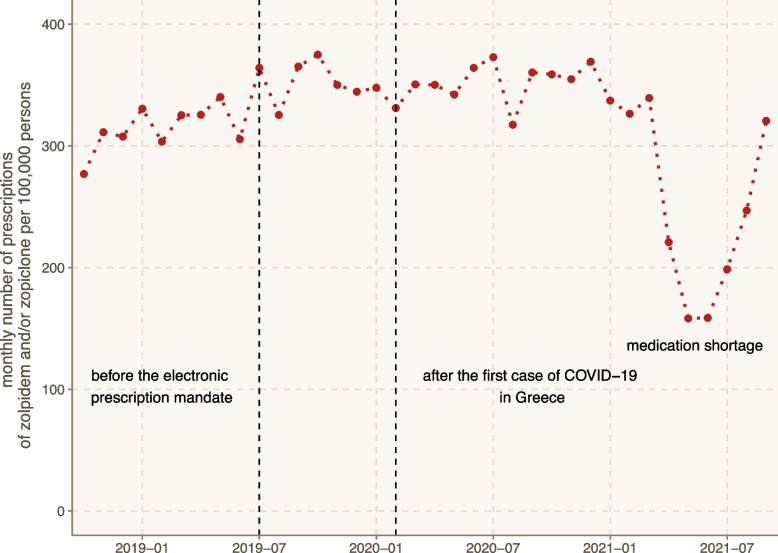
Monthly trends of prescriptions for Z-drugs in Greece. The figure presents the monthly number of prescriptions standardized per 100,000 persons within the three-year investigated timeframe (01.10.2018–01.10.2021). The dashed lines indicate important dates, namely the electronic prescription mandate (07.2019) and the first COVID-19 case in Greece (02.2020)

The monthly number of prescriptions per 100,000 persons for the whole period was relatively stable with a median of 334.2 IQR [310.4; 351.6], yet some time trends could be noted (Fig. 2). Before the electronic prescription mandate (October 2018 to July 2019), the monthly number of prescriptions per 100,000 persons had a median of 318.3 IQR [306.0; 329.3]. After the electronic prescription mandate, there was an increase of about 10% in the median monthly number of prescriptions, which remained relatively stable from July 2019 up to March 2021 (Mann–Whitney U = 27, *p*-value < 0.001).

However, after March 2021, there was an unexpected medication shortage during the COVID-19 pandemic, which was reflected by a rapid decrease and a subsequent increase in the monthly number of prescriptions.

## Discussion

Our analysis was the first to investigate the prescription characteristics and trends of Z-drugs in Greece. We analyzed 1,229,842 prescriptions for Z-drugs (corresponding to 156,554 unique patients), which were registered to the Greek nationwide prescription database within a three-year time period (2018–2021).

We found that about 1% of the Greek population was prescribed at least once a Z-drug (mostly zolpidem) over the course of a year. The prevalence was higher in females and almost exponentially increased with the age, i.e., about two thirds of the patients were older adults ≥ 65 years old. Patients had often other co-prescribed psychotropic medications (e.g., benzodiazepines in about a third of the patients, antidepressants and antipsychotics in about a tenth and twentieth respectively) and psychiatric disorders were also present, mainly anxiety and depressive disorders. Our analysis also indicated that the prevalence of Z-drug prescriptions increased in 2020, which coincided with the start of the COVID-19 pandemic and an associated increase in insomnia [3, 15]. This increase was particularly notable in younger populations, while a decrease was even observed among those aged over 85 years (eAppendix-3).

Our findings were consistent with previous studies from other countries, reporting similar patterns of Z-drug use and prescription characteristics [7–11]. A concerning trend was the substantial prescribing of Z-drugs among older adults, despite the potentially elevated risk of side-effects such as cognitive impairment, confusion, falls, fractures, and pneumonia [4, 16]. Although this finding may reflect a higher prevalence of insomnia in older adults, it highlights the importance of regularly evaluating the risk–benefit profile of Z-drugs and considering safer alternative interventions, especially for this vulnerable population [17]. Efforts should be made to increase the implementation of efficacious psychosocial and behavioral interventions, such as CBT-I, although their availability and accessibility are generally poor [4, 18]. Moreover, shared decision-making between patients and physicians should be implemented to evaluate the benefits and harms of currently available medications [4, 5, 19]. However, further research is needed, as there is limited randomized evidence on the efficacy and safety of pharmacological and non-pharmacological interventions for insomnia in older adults [20].

Furthermore, our study found that more than two-thirds of the patients were exclusively prescribed by internists and general practitioners, likely reflecting the higher prevalence of insomnia in primary care settings and the distribution of medical specialists in Greece. However, general practitioners may often encounter challenges in optimally diagnosing and treating insomnia as well as the frequently coexisting major mental disorders [18, 21]. Notably, almost half of the patients with anxiety or depressive disorders did not receive a prescription for an antidepressant or anxiolytic. This situation was more frequently observed in prescriptions by medical specialties other than psychiatrists or neurologists. However, it is worth noting that our study may have underestimated the extent of co-prescription for medications, since we did not have access to data for prescriptions that did not include Z-drugs (e.g., patients may have been prescribed psychotropic medications separately from their Z-drug prescription).

Another interesting finding was the increase of about 10% in the monthly number prescriptions after the electronic prescription mandate (Fig. 2), indicating that a number of prescriptions for Z-drugs may not have been registered in the prescription database before the mandate. Therefore, this finding can provide an additional explanation to the observed increase in the prevalence of Z-drug prescriptions during 2020 compared to 2019, considering that the mandate was only active for approximately half of the 2019 period. Moreover, this policy could facilitate the surveillance of prescriptions for drugs with abuse and dependence potential, which could be further enhanced by building an infrastructure of Addictovigilance [22]. Additional policy changes may also be useful in reducing Z-drugs prescriptions [23], but their potential drawbacks should be taken into consideration, including the increased complexity in clinical practice and the possibility of patients switching from Z-drugs to benzodiazepines or other substances with abuse potential [24, 25].

Our analysis has certain limitations. First, there was insufficient information about the prescribed dose that precluded any further investigation. While the data included the number of Z-drug boxes, there were no further details regarding the prescribed dose. Therefore, a more elaborate analysis considering also estimations of continuous and long-term use of Z-drugs was not possible. The relationship between the prescribed dose and sex would also be important given that women have a lower clearance of zolpidem. Accordingly, an FDA warning recommended the use of lower doses of zolpidem in women in order to avoid residual morning sleepiness and impairments in psychomotor performance [26], yet the evidence basis of this warning has been questioned [27]. Second, data for prescriptions that did not contain Z-drugs were not obtained. Therefore, we could not conduct a more comprehensive analysis of the comorbidities and concomitant medications. Accordingly, although our dataset included information about ICD-10 codes, it was not possible with the current data to differentiate between whether a diagnosis was the reason for prescribing Z-drugs or a comorbidity. Third, it is important to acknowledge that our method of identifying diagnoses based on at least one prescription with the corresponding ICD-10 may have led to an overestimation of the prevalence of certain conditions [28, 29]. Nevertheless, there is no universally accepted approach, and we preferred to prioritize a higher sensitivity given that we did not have access to all the prescriptions of the patients. Fourth, the available data covered a limited timeframe of three years, during which the COVID-19 pandemic and medication shortages occurred, potentially influencing the prescription patterns. However, there were no substantial changes in the monthly number of prescriptions, except during the period of medication shortage. Finally, we acknowledge that we did not register a detailed protocol for this study, which had, however, a descriptive and explorative nature.

## Conclusions

In conclusion, this was the first study that utilized the Greek national prescription database in order to explore the Z-drug prescribing. The results indicated suggesting that about 1% of the Greek population were prescribed Z-drugs at least once over the course of a year. The prevalence was higher and substantial in older adults, as well as there were considerable rates of psychiatric comorbidity and concomitant psychotropics. The majority of the prescriptions were conducted by internists and general practitioners. Therefore, our findings underscore the high frequency of Z-drug prescriptions in older adults, emphasizing the importance of regular monitoring of their benefit-risk ratio in this vulnerable population. However, due to the limitations inherent to medical claims databases and the insufficient information about the dose, duration of use and concurrent prescribed medications, further research is warranted to evaluate the potential abuse and misuse of Z-drugs in Greece.

## Supplementary Information

**Additional file 1: eAppendix-1.** Descriptive characteristics of prescriptions. **eAppendix-2.** Differences between prescriptions by psychiatrists/neurologists vs. other specialties.** eAppendix-3.** Prevalence estimates of Z-drug prescriptions stratified by age and sex in 2019 and 2020.

## Data Availability

The dataset used in this analysis was requested from the Greek e-Government Center for Social Security Services (IDIKA S.A.) and the Greek Ministry of Health (https://www.moh.gov.gr/). The data are not readily available because they are property of the Greek Ministry of Health. Requests to access the dataset should be directed to sitecontact@moh.gov.gr.

## Abbreviations

95%CI: 95% Confidence intervals
ATC code: Anatomical Therapeutic Chemical code
CBT-I: Cognitive Behavioral Therapy for Insomnia
ICD-10: International Classification of Diseases version 10
IDIKA: Greek e-Government Center for Social Security Services
OR: Odds ratios
