# Supplementary material for: Prescribing Z-drugs in Greece: an analysis of the national prescription database from 2018 to 2021
Source: BMC Psychiatry. 2023 May 26;23:370. doi: 10.1186/s12888-023-04793-x (PMC10214344; doi:10.1186/s12888-023-04793-x)
Supplement: Supplementary file 1 — Additional file 1: eAppendix-1. Descriptive characteristics of prescriptions. eAppendix-2. Differences between prescriptions by psychiatrists/neurologists vs. other specialties. eAppendix-3. Prevalence estimates of Z-drug prescriptions stratified by age and sex in 2019 and 2020. [file 12888_2023_4793_MOESM1_ESM.pdf]

eAppendix

Z-drug prescribing in Greece: an analysis of the national prescription database from 2018 to 2021

Contents

Contents *2*

eAppendix-1: Descriptive characteristics of prescriptions *3*

eAppendix-1.1.: ICD-10 diagnoses in prescriptions (frequency >1%) **3**

eAppendix-1.2.: ICD-10 diagnoses in prescriptions that did not have a sleep disorder (ICD-10 G47 or F51) (frequency >1%) **4**

eAppendix-1.3.: Co-prescribed medications in prescriptions (frequency >1%) **5**

eAppendix-1.4.: Region of prescriptions **6**

eAppendix-2.: Differences between prescriptions by psychiatrists/neurologists vs. other specialties *8*

eAppendix-3: Prevalence estimates of Z-drug prescriptions stratified by age and sex in 2019 and 2020 .... *10*

eAppendix-1: Descriptive characteristics of prescriptions

eAppendix-1.1.: ICD-10 diagnoses in prescriptions (frequency >1%)

Any deviations from the data presented in the manuscript can be attributed to the presentation of the specific categories of ICD-10 codes below (e.g., F51.0 and F51 were reported separately below).

ICD10	Description	Number of prescriptions (N=1229842)	Frequency (%)
G47	Sleep disorders	284408	23.13%
F51.0	Nonorganic insomnia	174779	14.21%
F41.9	Anxiety disorder, unspecified	114855	9.34%
F41.2	Mixed anxiety and depressive disorder	89384	7.27%
F41.1	Generalized anxiety disorder	83644	6.80%
G47.0	Insomnia	60490	4.92%
F51	Nonorganic sleep disorders	46750	3.80%
F51.9	Sleep disorder not due to a substance or known physiological condition, unspecified	42871	3.49%
F41	Other anxiety disorders	37928	3.08%
I10	Essential (primary) hypertension	29723	2.42%
F32.9	Major depressive disorder, single episode, unspecified	22117	1.80%
G47.9	Sleep disorder, unspecified	21362	1.74%
F32	Major depressive episode	20019	1.63%
F33	Major depressive disorder, recurrent	18410	1.50%
T88.7	Unspecified adverse effect of drug or medicament	16371	1.33%
E78.0	Pure hypercholesterolemia	15830	1.29%
E11	Type 2 diabetes mellitus	13779	1.12%
F31	Bipolar disorder	13765	1.12%
I11.9	Hypertensive heart disease without heart failure	13104	1.07%

eAppendix-1.2.: ICD-10 diagnoses in prescriptions that did not have a sleep disorder (ICD-10 G47 or F51) (frequency >1%)

The ICD-10 codes refer to the ones reported in the prescriptions of Z-drugs. It was unclear if they were used as indication for the Z-drugs or other co-prescribed medications. It should also be noted that we did not have access to data for all prescriptions, i.e., prescriptions that did not contain Z-drugs, thus, the frequency of co-prescription with other medications may be underestimated.

ICD-10	Description	Number of prescriptions (N=587284)	Frequency (%)
F41.9	Anxiety disorder, unspecified	102177	17.40%
F41.2	Mixed anxiety and depressive disorder	80768	13.75%
F41.1	Generalized anxiety disorder	75884	12.92%
F41	Other anxiety disorders	34258	5.83%
F32.9	Major depressive disorder, single episode, unspecified	19855	3.38%
F32	Depressive episode	18286	3.11%
I10	Essential (primary) hypertension	18108	3.08%
F33	Major depressive disorder, recurrent	17400	2.96%
F31	Bipolar disorder	13284	2.26%
F32.8	Other depressive episodes	11005	1.87%
F20	Schizophrenia	10924	1.86%
T88.7	Unspecified adverse effect of drug or medicament	10287	1.75%
F34.1	Dysthymic disorder	10167	1.73%
I11.9	Hypertensive heart disease without heart failure	9722	1.66%
E78.0	Pure hypercholesterolemia	9665	1.65%
F29	Unspecified psychosis not due to a substance or known physiological condition	9586	1.63%
Z73.3	Stress, not elsewhere classified	8535	1.45%
E11	Type 2 diabetes mellitus	8369	1.43%
F09	Unspecified mental disorder due to known physiological condition	8203	1.40%
F39	Unspecified mood [affective] disorder	7410	1.26%

eAppendix-1.3.: Co-prescribed medications in prescriptions (frequency >1%)

It should be noted that we did not have access to data for all prescriptions, i.e., prescriptions that did not contain Z-drugs, thus, the frequency of co-prescription with other medications may be underestimated.

ATC code	Description	Number of prescriptions (N=1229842)	Frequency (%)
None	No other co-prescribed medications	593019	48.22%
N05BA08	bromazepam	156881	12.76%
N05BA12	alprazolam	142370	11.58%
N02BE51	paracetamol	92065	7.49%
N05BA06	lorazepam	53293	4.33%
C10AA05	atorvastatin	41188	3.35%
A02BC01	omeprazole	32087	2.61%
B01AC04	clopidogrel	21943	1.78%
N05BA01	diazepam	21862	1.78%
C10AA01	simvastatin	21222	1.73%
B01AC06	acetylsalicylic acid	20978	1.71%
N06AB04	citalopram	20073	1.63%
H03AA01	levothyroxine sodium	19954	1.62%
N05AH04	quetiapine	19012	1.55%
N06AB10	escitalopram	17781	1.45%
N06AX11	mirtazapine	17329	1.41%
A10BA02	metformin	17201	1.40%
C08CA01	amlodipine	16546	1.35%
C03CA01	furosemide	16019	1.30%
C09DA04	Irbesartan and diuretics	14700	1.20%
M04AA01	allopurinol	14555	1.18%
C07AB12	nebivolol	13900	1.13%
A02BC02	pantoprazole	13448	1.09%
C07AG02	carvedilol	13437	1.09%
N06AX16	venlafaxine	12804	1.04%

eAppendix-1.4.: Region of prescriptions

The prescriptions per region in Greece and their percentage to the total number of prescriptions (1229842) within the 3-year period are presented below. The percentage of prescriptions per region ("observed") was compared with the percentage of the population of the region (using the average of the population estimates for the 3-year period, total 10715740) ("expected"). We did not statistically test the differences (e.g., with a one-proportion z-test), since even small differences between observed and expected proportions would have been highly significant due to the large sample size.

Region	Number of prescriptions in the region within the 3-year period (N=1229842)	% Prescriptions	Population of the region (average within the 3-year period) (N=10,715,740)	% Population	Differences of the two proportions
ATTIKI	411456	33.46%	3743582	34.94%	-1.48%
THESSALONIKI	111833	9.09%	1102760	10.29%	-1.20%
LARISSA	27613	2.25%	280383.5	2.62%	-0.37%
ACHAEA	25837	2.10%	298051.3	2.78%	-0.68%
MAGNESIA	23715	1.93%	202684.8	1.89%	0.04%
SERRES	22955	1.87%	166686.5	1.56%	0.31%
DODEKANISA	20882	1.70%	218258.8	2.04%	-0.34%
HERAKLION	18123	1.47%	314208.8	2.93%	-1.46%
PIERIA	17730	1.44%	131773.5	1.23%	0.21%
AETOLIA-ACARNANIA	17709	1.44%	199764.5	1.86%	-0.42%
IOANNINA	17682	1.44%	167577.8	1.56%	-0.13%
CHANIA	17269	1.40%	159691	1.49%	-0.09%
LESBOS	17049	1.39%	115858.5	1.08%	0.31%
EYBOEA	15883	1.29%	212827.5	1.99%	-0.69%
IMATHIA	15829	1.29%	141403.8	1.32%	-0.03%
PELLA	15826	1.29%	136753.5	1.28%	0.01%
PHTIOTIS	15174	1.23%	158972.5	1.48%	-0.25%
KAVALA	15147	1.23%	133041.3	1.24%	-0.01%
MESSENIA	14329	1.17%	157826.5	1.47%	-0.31%

CORFU	13236	1.08%	101355.3	0.95%	0.13%
CORINTH	12532	1.02%	147479.5	1.38%	-0.36%
XANTHI	12481	1.01%	111442.3	1.04%	-0.03%
CYCLADES	12385	1.01%	126805.5	1.18%	-0.18%
KOZANI	12125	0.99%	139516	1.30%	-0.32%
KILKIS	11784	0.96%	80269.5	0.75%	0.21%
ILIA	11553	0.94%	155282.8	1.45%	-0.51%
VIOTIA	11069	0.90%	122348	1.14%	-0.24%
EVROS	11062	0.90%	147087.8	1.37%	-0.47%
DRAMA	11018	0.90%	96665.75	0.90%	-0.01%
KARDITSA	10796	0.88%	104724.5	0.98%	-0.10%
TRIKALA	10751	0.87%	128614.3	1.20%	-0.33%
CHALKIDIKI	9775	0.79%	110510.5	1.03%	-0.24%
RODOPI	8167	0.66%	110367	1.03%	-0.37%
ARGOLIDA	8017	0.65%	96317.25	0.90%	-0.25%
ΛΑΚΩΝΙΑ	7790	0.63%	90271.75	0.84%	-0.21%
RETHYMNO	6268	0.51%	87653	0.82%	-0.31%
SAMOS	6103	0.50%	48566.5	0.45%	0.04%
ARKADIA	5926	0.48%	81278	0.76%	-0.28%
CHIOS	5587	0.45%	58301.5	0.54%	-0.09%
FLORINA	5526	0.45%	49334.5	0.46%	-0.01%
PREVEZA	4696	0.38%	57908.25	0.54%	-0.16%
ARTA	4446	0.36%	62558.25	0.58%	-0.22%
KEFALONIA	4379	0.36%	38631.75	0.36%	0.00%
THESPROTIA	4176	0.34%	45026.5	0.42%	-0.08%
LASITHI	3784	0.31%	73873.75	0.69%	-0.38%
KASTORIA	3447	0.28%	46354.75	0.43%	-0.15%
FOKIDA	3362	0.27%	42356.25	0.40%	-0.12%
ZAKYNTHOS	3086	0.25%	39682.25	0.37%	-0.12%
GREVENA	3037	0.25%	30532.75	0.28%	-0.04%
LEYKADA	2317	0.19%	23818.5	0.22%	-0.03%
EVRITANIA	1352	0.11%	18700.75	0.17%	-0.06%

eAppendix-2.: Differences between prescriptions by psychiatrists/neurologists vs. other specialties

Variables	Prescriptions by psychiatrists/neurologists (n=209175)	Prescription by other medical specialties (n=1020667)	Effect size of prescriptions by psychiatrists/neurologists vs. other medical specialties
Z-drug (zolpidem vs. only zopiclone)	85.4%	90.5%	OR=0.61, 95%CI: 0.60, 0.62
Age (years)	Median 69 years, IQR [57; 80]	Median 78 years, IQR [69; 86]	A=0.33 (medium)
Sex (% female)	65.9%	70.4%	OR=0.81, 95%CI: 0.80, 0.82
Region (% Attica/Thessaloniki)	62.1%	45.4%	OR=1.97, 95%CI: 1.95, 1.99
Sleep disorders (G47/F51)	27.9%	57.2%	OR=0.29, 95%CI: 0.29, 0.29
Specific sleep disorder diagnosis (F51 vs. G47)	There were 58343 prescriptions with F51 or G47, and F51 was indicated in 46.2%	There were 584124 prescriptions with F51 or G47, and F51 was indicated in 41.2%	OR=1.22, 95%CI: 1.20, 1.24
Anxiety or depressive disorders (ICD-10: F40/F41/F32/F33)	43.2%	33.5%	OR=1.51, 95%CI: 1.50, 1.52
Anxiety disorders (ICD-10: F40/F41)	25.2%	27.6%	OR=0.88, 95%CI: 0.87, 0.89
Depressive disorders (ICD-10: F32/F33)	18.5%	6.0%	OR=3.53, 95%CI: 3.49, 3.58
Co-prescribed anxiolytics (ATC: N05B)	45.7%	27.9%	OR=2.18, 95%CI: 2.16, 2.20
Co-prescribed antidepressants (ATC: N06A)	14.2%	6.3%	OR=2.45, 95%CI: 2.41, 2.49
Co-prescribed nonopioid analgesic (ATC: N02B)	2.8%	8.4%	OR=0.31, 95%CI: 0.30, 0.32
Co-prescribed antipsychotics (ATC: N05A)	8.8%	1.9%	OR=4.95, 95%CI: 4.85, 5.05
Co-prescribed antiepileptics (ATC: N03A)	3.3%	1.5%	OR=2.33, 95%CI: 2.26, 2.40
Co-prescribed opioid analgesics (ATC: N02A)	0.6%	1.9%	OR=0.29, 95%CI: 0.27, 0.30
Co-prescribed anxiolytic or antidepressants in prescriptions that had a diagnosis of anxiety or depressive disorder	63.1%	44.6%	OR=2.13, 95%CI: 2.10, 2.16

The effect-size for continuous variables was mean difference in case normality (tested with a t-test), otherwise the Vargha's and Delaney's A (tested with a Mann-Whitney

test). The effect-size for dichotomous variables was odds ratios (tested with a chi-squared). Due to the large sample size of the study, p-value were <0.001 in all cases. *We did not have access to data for all prescriptions, i.e., prescriptions that did not contain Z-drugs, thus, the frequency of co-prescription with other medications may be underestimated.

eAppendix-3: Prevalence estimates of Z-drug prescriptions stratified by age and sex in 2019 and 2020

The estimated overall prevalence standardized by age group and sex was 0.85% in 2019 and 0.89% in 2020.

Differences in the prevalence between the two years per age group and sex were compared with odds ratios (ORs) and their 95% confidence intervals (95%CI).

*The age group <20 years was not presented due to the small number of prescriptions. An increase in the prevalence of Z-prescriptions in this age group was generally noted between 2020 vs. 2019 (males: OR=2.14, 95%CI [0.87, 5.26], females: OR=3.60 95%CI [1.55, 8.3], both: OR=2.87, 95%CI [1.56, 5.27]).

		2019			2020			ORs for 2020 vs. 2019			
	Age group	Number of people receiving at least one prescription (N=91504)	Estimated population from the Hellenic Statistical Authority (N=10724599)	%	Number of people receiving at least one prescription (N=95537)	Estimated population from the Hellenic Statistical Authority (N=10718565)	%	OR	Lower boundary of the 95%CI	Upper boundary of the 95%CI	Weighted average of the prevalence of 2019-2020
females	20-24	61	265850	0.02%	95	266429	0.04%	1.55	1.13	2.14	0.03%
	25-29	132	279643	0.05%	181	274065	0.07%	1.40	1.12	1.75	0.06%
	30-34	210	305389	0.07%	308	298181	0.10%	1.50	1.26	1.79	0.09%
	35-39	361	382507	0.09%	468	367204	0.13%	1.35	1.18	1.55	0.11%
	40-44	756	404656	0.19%	998	402081	0.25%	1.33	1.21	1.46	0.22%
	45-49	1287	412102	0.31%	1582	408394	0.39%	1.24	1.15	1.34	0.35%
	50-54	2164	405972	0.53%	2661	413336	0.64%	1.21	1.14	1.28	0.59%
	55-59	3205	380563	0.84%	3762	379760	0.99%	1.18	1.12	1.24	0.92%
	60-64	4535	355526	1.28%	4982	362278	1.38%	1.08	1.04	1.12	1.33%

	65-69	5744	321158	1.79%	6265	323057	1.94%	1.09	1.05	1.13	1.86%
	70-74	7200	301680	2.39%	7850	307835	2.55%	1.07	1.04	1.11	2.47%
	75-79	8532	243441	3.50%	8777	240669	3.65%	1.04	1.01	1.07	3.58%
	80-84	10015	230859	4.34%	9892	231560	4.27%	0.98	0.96	1.01	4.30%
	85+	17218	216920	7.94%	15550	223368	6.96%	0.87	0.85	0.89	7.44%
	20-24	51	286472	0.02%	106	293177	0.04%	2.03	1.45	2.84	0.03%
	25-29	137	289329	0.05%	210	290611	0.07%	1.53	1.23	1.89	0.06%
	30-34	225	304326	0.07%	279	298257	0.09%	1.27	1.06	1.51	0.08%
	35-39	329	383663	0.09%	472	369591	0.13%	1.49	1.29	1.72	0.11%
	40-44	615	397530	0.15%	810	397708	0.20%	1.32	1.19	1.46	0.18%
	45-49	951	393951	0.24%	1083	391322	0.28%	1.15	1.05	1.25	0.26%
	50-54	1361	376235	0.36%	1587	384485	0.41%	1.14	1.06	1.23	0.39%
	55-59	1673	338020	0.49%	1967	337891	0.58%	1.18	1.10	1.26	0.54%
	60-64	2320	316380	0.73%	2704	320371	0.84%	1.15	1.09	1.22	0.79%
	65-69	2992	285694	1.05%	3286	285945	1.15%	1.10	1.05	1.15	1.10%
	70-74	3628	255713	1.42%	3974	260444	1.53%	1.08	1.03	1.13	1.47%
	75-79	3813	195153	1.95%	4145	195010	2.13%	1.09	1.04	1.14	2.04%
	80-84	4415	163766	2.70%	4513	164264	2.75%	1.02	0.98	1.06	2.72%
males	85+	7560	148889	5.08%	6990	154048	4.54%	0.89	0.86	0.92	4.80%
	20-24	112	552322	0.02%	201	559606	0.04%	1.77	1.41	2.23	0.03%
	25-29	269	568972	0.05%	391	564676	0.07%	1.46	1.25	1.71	0.06%
	30-34	435	609715	0.07%	587	596438	0.10%	1.38	1.22	1.56	0.08%
	35-39	690	766170	0.09%	940	736795	0.13%	1.42	1.28	1.56	0.11%
	40-44	1371	802186	0.17%	1808	799789	0.23%	1.32	1.23	1.42	0.20%
	45-49	2238	806053	0.28%	2665	799716	0.33%	1.20	1.14	1.27	0.31%
	50-54	3525	782207	0.45%	4248	797821	0.53%	1.18	1.13	1.24	0.49%
all	55-59	4878	718583	0.68%	5729	717651	0.80%	1.18	1.13	1.22	0.74%

	60-64	6855	671906	1.02%	7686	682649	1.13%	1.10	1.07	1.14	1.07%
	65-69	8736	606852	1.44%	9551	609002	1.57%	1.09	1.06	1.12	1.50%
	70-74	10828	557393	1.94%	11824	568279	2.08%	1.07	1.04	1.10	2.01%
	75-79	12345	438594	2.81%	12922	435679	2.97%	1.06	1.03	1.08	2.89%
	80-84	14430	394625	3.66%	14405	395824	3.64%	1.00	0.97	1.02	3.65%
	85+	24778	365809	6.77%	22540	377416	5.97%	0.87	0.86	0.89	6.37%
